# The risk of cardiovascular comorbidity in children with Behçet’s disease

**DOI:** 10.1093/rheumatology/kead505

**Published:** 2023-10-03

**Authors:** Selcan Demir, Ali Duzova, Tevfik Karagoz, Berna Oguz, Hayrettin Hakan Aykan, Ozlem Satirer, Erdal Sag, Seza Ozen, Yelda Bilginer

**Affiliations:** Department of Pediatric Rheumatology, Eskisehir Osmangazi University Medical Faculty, Eskisehir, Turkey; Department of Pediatric Nephrology, Hacettepe University Medical Faculty, Ankara, Turkey; Department of Pediatric Cardiology, Hacettepe University Medical Faculty, Ankara, Turkey; Department of Pediatric Radiology, Hacettepe University Medical Faculty, Ankara, Turkey; Department of Pediatric Cardiology, Hacettepe University Medical Faculty, Ankara, Turkey; Department of Pediatrics, Hacettepe University Medical Faculty, Ankara, Turkey; Department of Pediatric Rheumatology, Hacettepe University Medical Faculty, Ankara, Turkey; Department of Pediatric Rheumatology, Hacettepe University Medical Faculty, Ankara, Turkey; Department of Pediatric Rheumatology, Hacettepe University Medical Faculty, Ankara, Turkey

**Keywords:** paediatric Behçet’s disease, cardiovascular comorbidity, premature atherosclerosis, hypertension, arterial stiffness, intima-media thickness, twenty-four-hour ambulatory blood pressure monitoring, ABPM, transthoracic echocardiography

## Abstract

**Objective:**

Patients with Behçet’s disease (BD) may experience long-term morbidity caused by various forms of cardiovascular disease. This study aimed to assess the risk for cardiovascular comorbidity in paediatric BD patients with and without vascular involvement, independent of the contribution of traditional risk factors.

**Methods:**

Paediatric patients classified as having BD according to the 2015 Peadiatric BD (PEDBD) criteria were included in the study. Twenty-four-hour ambulatory blood pressure monitoring (ABPM), transthoracic echocardiography, and carotid intima-media thickness (cIMT) measurements were performed. Patients with an active disease or those who have other known risk factors for cardiovascular disease were not included in the study.

**Results:**

Thirty-one children and adolescents with paediatric BD (16 female, 51.6%; F/M: 1.06) were enrolled in the study. Among the BD patients, 10 patients (34.4%) had abnormal ABPM. Carotid IMT values, mean arterial pressure, systolic and diastolic blood pressure by ABPM and the prevalence of abnormal ABPM, non-dipping, and ambulatory hypertension were similar between patients with and without vascular involvement. The echocardiography measurements showed that BD patients with vascular involvement had a significantly higher velocity and velocity time integral of the left ventricle outflow tract, which may indicate increased stiffness of the aorta.

**Conclusion:**

Paediatric BD patients with vascular involvement may tend to have more cardiovascular risk factors. However, cardiovascular assessment should be considered in all BD patients, regardless of the involved systems. We suggest that ABPM may accurately define hypertension and cardiovascular risk in BD.

Rheumatology key messages

- Paediatric Behçet’s disease (BD) patients with vascular involvement may tend to have more cardiovascular risk factors.
- BD patients should be evaluated for cardiovascular comorbidities, regardless of the involved systems.
- ABPM may accurately identify hypertension and increased cardiovascular risk in BD.

## Introduction

Behçet’s disease (BD) is a multigenic autoinflammatory disorder characterized by recurrent mucocutaneous, ocular, musculoskeletal, vascular, gastrointestinal and CNS manifestations [1]. Unlike other systemic vasculitis, BD can affect any type and size of blood vessels [2]. Although BD patients are diagnosed more frequently in the second or third decade of life, the first symptoms appear under the age of 16 years in 4–26% of patients [3]. Due to the rarity and heterogeneity of BD during childhood, the diagnosis and management are challenging.

The overall mortality rate of patients with BD is significantly higher among patients under 25 years of age and in the early disease course. Furthermore, vascular involvement is one of the major causes of morbidity and mortality and occurs in up to 40% of patients with BD [4].

Considering the increased inflammatory nature of BD compared with other forms of vasculitis, it is expected that recurrent inflammation attacks will affect the endothelium and contribute to the development of atherosclerosis chronically. Recent studies showed that increased carotid intima-media thickness (cIMT) was significantly associated with endothelial cell dysfunction (ECD), which is regarded as the early marker of atherogenesis [5, 6]. ECD has also been shown in patients with BD [7–10]. However, the contribution of accelerated and early atherosclerosis to cardiovascular comorbidity of BD needs to be clarified. Cardiac involvement in BD has been reported between 2.4 and 6.4% of patients and includes coronary artery disease, as well [4, 11, 12]. Cardiovascular disease affects mostly younger BD patients and is often manifested in the form of myocardial infarction or angina [13]. A prospective study on BD patients who had no history of cardiovascular disease reported that BD patients had intrinsic left ventricular dysfunction on speckle tracking echocardiography, despite having no apparent abnormality on routine echocardiography [14]. It is still under debate to what extent BD increases the cardiovascular disease risk, especially in individuals without other traditional risk factors such as obesity, metabolic syndrome, or smoking. The increased burden of cardiovascular comorbidity in SLE patients has already been well documented [15]; traditional atherosclerotic risk factors contribute to increased risk but cannot fully explain the accelerated cardiovascular disease in SLE [16]. This risk is even more pronounced among younger SLE patients [17].

In fact, vascular BD is mostly seen in younger patients, and it is not clear whether BD patients with vascular involvement are at greater risk for future cardiovascular disease. In this study, we aimed to evaluate the cardiovascular assessment of paediatric BD patients with and without vascular involvement. We evaluated 31 paediatric BD patients with 24-h ambulatory blood pressure monitoring (ABPM), transthoracic echocardiography, and cIMT to determine whether there is an increased risk of cardiovascular comorbidity in the paediatric population with vascular BD.

## Patients and methods

### Study population

Thirty-one BD patients who were followed at a paediatric rheumatology outpatient clinic between January 2017 and December 2019 were prospectively enrolled into the study. Patients (<16 years of age at disease onset and diagnosis) were classified as having BD according to the Pediatric Behçet’s Disease (PEDBD) classification criteria [18]. Patients with a history of arterial occlusions, and/or stenosis, and/or aneurysms and/or venous thrombosis formed the BD patient group with vascular involvement.

Current disease activity during the assessments was calculated for each patient using the BD activity form [19]. Patients with an active organ and/or system involvement (oral ulcer, genital ulcer, skin lesions, uveitis, retinal vasculitis, thrombophlebitis, arthritis, gastrointestinal involvement, neurologic involvement) and patients who had other known risk factors for cardiovascular disease [such as obesity (BMI > 30), hypertension, diabetes mellitus, or metabolic syndrome] were excluded.

When the patients came for routine visits, a physical examination and laboratory tests (complete blood count, serum creatinine, lipid profile, acute phase reactants, and urine test) were performed. During the same week, cIMT measurement, echocardiography and 24-h ABPM were also performed. Ethical approval was obtained from the Hacettepe University Faculty of Medicine Institutional Review Board. Written informed consent was obtained from the parents of all subjects involved in the study.

### Twenty-four-hour ambulatory blood pressure monitoring

Twenty-four-hour ABPM was performed in 29 patients; Spacelabs ABPM devices (Model no: 90207–30) were used. Measurements were performed every 15–20 min during daytime (waking hours) and every 30 min during the night (sleep periods). The mean systolic BP (SBP), diastolic BP (DBP) and arterial pressure (MAP) levels and load (percentage of readings above the ambulatory 95th percentile by sex and age) were calculated for the 24-hour period. Daytime was defined as from 08.00 a.m. to 08.00 pm, and nighttime was defined as from midnight to 06.00 a.m. S.D. scores for SBP, DBP and MAP were determined according to the standard values adjusted by age and gender [20]. S.D. score indicates how many S.D.s an observation is above or below the mean/median: S.D. score = (observed value – mean/median value of the reference population)/(S.D. value of reference population). The presence of SBP or DBP or MAP being equal to or greater than the 95th percentile was defined as hypertension. Dipping was defined as the percentage decline in mean systolic or diastolic levels from daytime to nighttime [100 × (mean daytime – mean nighttime)/(mean daytime)]; non-dipping was defined as a dipping level of <10%.

### Carotid intima-media thickness measurements

The intima-media thickness of a vessel is the distance from the lumen–intima interface to the media adventitia interface. US examination was performed to measure the cIMT of the right and left common carotid arteries. To avoid inter-observer variability, all cIMT measurements were performed by the same expert, who was unaware of the clinical characteristics of the patients. Patients were scanned in the supine position and with the neck rotated to the opposite side of examination by using an 8-MHz annular array US imaging system (Siemens Acuson P50 Ultrasound system, Siemens Medical Solution USA, Inc. Mountain View, California, USA). The cIMT was measured at 10 mm proximal to the distal end of the common carotid artery (CCA) in a straight segment (at least 10 mm long) from the far wall. Five measurements were obtained and recorded in millimetres (mm) for the right and left common carotid arteries, then the arithmetic mean was calculated for each side. cIMT S.D.s adjusted for age, height and sex were calculated using reference values [21].

### Transthoracic echocardiographic assessments

All patients underwent standard M-mode, 2-dimensional and Doppler transthoracic echocardiographic assessments. Echocardiographic evaluations were performed using a GE Vivid E9 with XD clear or GE Vivid S5 (GE Healthcare, Horten, 70 Norway) with a 5 or 6 MHz matrix transducer probe. All assessments were performed by the same examiner, who was blinded to the clinical characteristics of the patients. Cardiac chamber quantification, Doppler echocardiography and M-mode echocardiographic measurements (left atrial diameter, aortic diameter, interventricular septal thickness, posterior wall thickness, left ventricular internal dimensions, tricuspid annular plane systolic excursion) were performed according to the established standards of the American Society of Echocardiography [22]. Left ventricular mass was calculated using the formula given by Devereux *et al.* according to the American Society of Echocardiography guidelines: left ventricular mass (g) = 0.81 [1.04 (interventricular septum thickness + posterior wall thickness + left ventricular internal dimension)^3^ − (left ventricular internal dimension)^3^] + 0.06. The left ventricular mass index was derived by dividing the left ventricular mass in grams by the patient’s body surface area. Relative wall thickness was defined as the ratio of the end-diastolic left ventricular posterior wall thickness to the left ventricular internal dimension [(2 × posterior wall thickness)/(left ventricular internal dimension)].

### Statistical analysis

Statistical analyses were performed using the SPSS software v. 23 (IBM, Armonk NY). The variables were investigated using visual (histogram, probability plots) and analytic methods (Kolmogorov–Smirnov/Shapiro–Wilk’s test) to determine whether or not they were normally distributed. Descriptive analyses were presented using means and S.D.s for normally distributed variables and medians and interquartile range (IQR) for the non-normally distributed and ordinal variables. χ^2^ analysis and Student’s *t* test were used for the comparison of two parametric variables, while the Mann–Whitney *U* test was used in the presence of non-parametric data. Correlations between cIMT values and various continuous variables were investigated using Pearson and Spearman correlation analyses. *P*-values of <0.05 were accepted as significant.

## Results

### Demographics and clinical characteristics

Thirty-one patients [16 female (51.6%; female:male ratio 1.06)] diagnosed with childhood-onset BD were included in the study. All the patients (*n* = 31, 100%) were Turkish. The mean age at disease onset and at the time of diagnosis was 8.79 ± 4.31 years and 11.70 ± 3.25 years, respectively. Based on the cumulative disease characteristics, oral ulcer was the most common clinical finding (100%), followed by skin involvement (77.4%, *n* = 24), arthritis (54.8%, *n* = 17) and genital ulcers (48.4%, *n* = 14). Nine patients (29%) had a history of ocular involvement, and six patients (19.4%) had vascular involvement. The most common form of vascular involvement was sinus vein thrombosis (*n* = 3, 50%), followed by femoral vein involvement (2/6, 33.3%), descending abdominal aorta involvement (*n* = 1, 16.6%), iliac artery involvement (*n* = 1, 16.6%), popliteal artery involvement (*n* = 1, 16.6%) and brachial vein involvement (*n* = 1, 16.6%). The HLA-B51 allele was carried by 19 patients (61.3%), and 8 patients (25.8%) had a positive pathergy test.

We compared the patient demographics and clinical characteristics of BD patients based on the presence of vascular involvement. There was no statistical difference based on gender or age between the two groups. Patients with vascular involvement were more likely to have CNS involvement (16.6% parenchymal) (50%, *n* = 3 *vs* 8%, *n* = 2, *P* = 0.038) and had received more immunosuppressant therapy than those without (100%, *n* = 6 *vs* 36%, *n* = 9, *P* = 0.005). When we compared the laboratory levels at the time of study recruiting, the levels of very-low-density lipoprotein (VLDL) [21.50 mg/dl (17–27) *vs* 13 mg/dl (10–20), *P* = 0.026], triglycerides [105.5 mg/dl (87–135) *vs* 66 mg/dl (54–94), *P* = 0.021] and CRP [1.1 mg/dl (0.5–1.7) *vs* 0.4 mg/dl (0.2–0.7), *P* = 0.047] were higher and the level of high-density lipoprotein (HDL) [40.50 mg/dl (31–45) *vs* 46 (44–57), *P* = 0.035] was lower in patients with vascular involvement compared with the patients without vascular involvement. With respect to the other parameters [including leucocytes, haemoglobin, platelet count, serum homocysteine, total cholesterol, low-density lipoprotein (LDL) levels, and ESR] there were no statistically significant differences between the groups. The demographic and clinical characteristics are summarized in Table 1.

**Table 1.** Patients’ demographics, and clinical and laboratory characteristics according to vascular involvement

Characteristics	Vascular involvement		*P*-value
	Yes (*n* = 6)	No (*n* = 25)	
Gender, F/M	4/2	12/13	0.654
Age at disease onset, years	12.23 (7.84–13.72)	9.18 (5.88–11.62)	0.516
Age at diagnosis, years	12.45 (7.84–13.72)	12.21(9.88–13.88)	0.920
Disease duration, months	70.98 (63.57–148.80)	75.47 (46.62–112.10)	0.484
Delay in the diagnosis, months	11.98 (00–28.98)	17.97 (5.03–88.97)	0.342
Age at the time of study, years	18.06 (17.18–20.06)	16.84 (11.50–18.35)	0.147
Oral ulcer, *n* (%)	6 (100.0%)	25 (100.0%)	–
Genital ulcer, *n* (%)	3 (50.0%)	12 (48.0%)	1.0
Skin involvement, *n* (%)	4 (66.7%)	20 (80.0%)	0.596
Erythema nodosum, *n* (%)	2 (33.3%)	12 (48.0%)	0.664
Pathergy positivity, *n* (%)	2 (33.3%)	6 (24.0%)	0.634
Ocular involvement, *n* (%)	2 (33.3%)	7 (28.0%)	1.0
Arthritis, *n* (%)	2 (33.3%)	15 (60.0%)	0.370
CNS involvement, *n* (%)	**3 (50.0%)**	2 (8.0%)	**0.038**
HLA-B51 positivity, *n* (%)	4 (66.7%)	15 (60.0%)	1.0
Leucocytes/mm^3^	5650 (4700–6200)	6400 (5500–7700)	0.176
Haemoglobin, g/dl	13.25 (12.60–14.80)	13.50 (12.80–14.90)	0.822
Platelets/mm^3^	213 000 (186 000–270 000)	252 000 (232 000–281 000)	0.089
Total cholesterol, mg/dl	123 (118–148)	147 (133–168)	0.162
HDL, mg/dl	**40.50 (31–45)**	46 (44–57)	**0.035**
LDL, mg/dl	83 (79–101)	89 (82–104)	0.466
VLDL, mg/dl	**21.50 (17–27)**	13 (10–20)	**0.026**
Triglycerides, mg/dl	**105.5 (87–135)**	66 (54–94)	**0.021**
Homocysteine, mmol/l	13.25 (9–21)	15 (9.7–16.5)	0.953
ESR, mm/h	17.5 (2–24)	8 (4.12)	0.340
CRP, mg/dl	**1.1 (0.5–1.7)**	0.4 (0.2–0.7)	**0.047**
CS, *n* (%)	6 (100%)	19 (76%)	0.181
Immunosuppressive, *n* (%)	6 (100%)	9 (36%)	0.005
Colchicine, *n* (%)	4 (66.7%)	22 (88%)	0.202

Continuous parameters are presented as median (interquartile range, IQR). Bold text highlights statistically significant values. ^a^All the laboratory tests were on the same day with cIMT. HDL: high-density lipoprotein, LDL: low-density lipoprotein, VLDL: very low-density lipoprotein.

### Ambulatory blood pressure monitoring

Twenty-four-hour ABPM was performed in 29 BD patients. Among them, 10 patients (34.4%) had abnormal ABPM. Twenty-four-hour, daytime and nighttime MAP S.D. scores, SBP S.D. scores and DBP S.D. scores were statistically similar between the two groups. (Table 2). Seven patients were non-dippers (24.1%), and hypertension was detected in 5 BD (17.2%, 5/29) patients. Abnormal ABPM frequency (50%, *n* = 3 *vs* 30.4%, *n* = 7, *P* = 0.633), the frequency of non-dippers (33.3%, *n* = 2 *vs* 21.7%, *n* = 5, *P* = 0.612) and the frequency of hypertension (33.3%, *n* = 2 *vs* 13%, *n* = 3, *P* = 0.269) also did not differ significantly between patients with and without vascular involvement, respectively.

**Table 2.** Twenty-four-hour ambulatory blood pressure monitoring findings of patients with Behçet’s disease according to vascular involvement

Parameters	Vascular involvement		*P*-value
	Yes (*n* = 6)	No (*n* = 23)	
24-h			
MAP, mm	87.0 ± 6.5	84.2 ± 4.9	0.130
SBP, mm	120.2 ± 13.0	120.2 ± 13.0	0.089
DBP, mm	69.5 ± 3.0	68.7 ± 4.3	0.607
HR, bpm	78.2 ± 6.8	83.3 ± 8.6	0.138
MAP S.D. score	0.55 ± 0.99	0.21 ± 0.73	0.196
SBP S.D. score	0.34 ± 1.68	–0.42 ± 0.80	0.196
DBP S.D. score	0.27 ± 0.54	0.19 ± 0.75	0.746
HR S.D. score	0.14 ± 0.78	0.48 ± 0.82	0.294
Day-time			
MAP, mm	91.2 ± 6.8	88.7 ± 5.7	0.144
SBP, mm	123.8 ± 13.1	117.04 ± 8.1	0.095
DBP, mm	74.0 ± 4.6	73.43 ± 5.0	0.705
HR, bpm	84.2 ± 7.7	88.4 ± 9.6	0.281
MAP S.D. score	0.42 ± 0.94	0.17 ± 0.78	0.225
SBP S.D. score	0.19 ± 1.57	–0.41 ± 0.85	0.187
DBP S.D. score	0.10 ± 0.80	0.09 ± 0.81	0.829
HR S.D. score	0.27 ± 0.78	0.42 ± 0.84	0.467
Night-time			
MAP, mm	76.8 ± 7.6	73.5 ± 4.3	0.291
SBP, mm	109.5 ± 13.2	100.7 ± 6.6	0.118
DBP, mm	58.7 ± 3.3	57.0 ± 3.8	0.317
HR, bpm	65.3 ± 5.1	71.6 ± 9.1	0.105
MAP S.D. score	0.78 ± 1.33	0.24 ± 0.64	0.419
SBP S.D. score	0.57 ± 1.81	−0.43 ± 0.62	0.282
DBP S.D. score	0.49 ± 0.59	0.19 ± 0.66	0.319
HR S.D. score	0.03 ± 0.49	0.51 ± 0.86	0.146
Abnormal ABPM, *n* (%)	3 (50.0)	7 (30.4%)	0.633
Non-dipper, *n* (%)	2 (33.3)	5 (21.7%)	0.612
Hypertension, *n* (%)	2 (33.3)	3 (13.0%)	0.269

ABPM: ambulatory blood pressure monitoring, DBP: diastolic blood pressure, HR: heart rate. MAP: mean arterial blood pressure, SBP: systolic blood pressure.

### Echocardiography

Table 3 summarizes the standard M-mode, 2-dimensional and Doppler echocardiographic findings for BD patients with and without vascular involvement. Patients with vascular involvement had a significantly higher left ventricle outflow Doppler velocity [1.25 ± 0.05 *vs* 1.10 ± 0.31 (*P* = 0.002)] and velocity time integral for the aorta [24.45 ± 1.50 *vs* 20.43 ± 3.53 (*P* = 0.006)].

**Table 3.** Echocardiography findings of patients with Behçet’s disease according to vascular involvement

Parameters	Vascular involvement		*P*-value
	Yes (*n* = 6)	No (*n* = 25)	
TAPSE, mm	19.60 ± 3.51	21.65 ± 3.31	0.216
TAPSE S.D. score	–2.25 ± 1.62	–0.80 ± 1.69	0.099
LVM, g	121 ± 30.01	114.12 ± 53.18	0.368
LVM S.D. score	–0.58 ± 0.93	–0.96 ± 2.12	0.653
LVMI, g/m^2^	70.33 ± 8.82	74.56 ± 21.53	0.438
LAtr diameter, cm	2.75 ± 0.30	2.94 ± 0.68	0.636
LAtr diameter S.D. score	0.40 ± 0.69	0.88 ± 0.94	0.169
Ao diameter, mm	2.86 ± 0.15	2.73 ± 0.32	0.211
Ratio of LAtr to Ao diameter	0.96 ± 0.1	1.06 ± 0.1	0.052
Relative wall thickness	0.39 ± 0.07	0.40 ± 0.11	0.960
IVST-D, cm	0.88 ± 0.13	0.88 ± 0.16	0.878
IVST-D S.D. score	0.53 ± 0.20	0.85 ± 0.51	0.121
IVST-S, cm	1.35 ± 0.15	1.28 ± 0.26	0.348
IVST-S S.D. score	1.00 ± 0.40	1.05 ± 0.74	0.940
LVID-D, cm	4.37 ± 0.41	4.16 ± 0.72	0.366
LVID-D S.D. score	–1.16 ± 1.35	–1.18 ± 1.63	0.337
LVID-S, cm	2.73 0.36	2.56 0.46	0.391
LVID-S S.D. score	–0.85 ± 1.61	–1.04 ± 1.85	0.617
LVPWT-D, cm	0.84 ± 0.13	0.82 ± 0.16	0.745
LVPWT-D S.D. score	0.74 ± 0.68	0.95 ± 0.69	0.51
LVPWT-S, cm	1.35 ± 0.19	1.22 ± 0.28	0.244
LVPWT-S S.D. score	–0.03 ± 0.49	–0.33 ± 1.20	0.764
Diastolic arterial pressure, mm	73.33 ± 7.53	71.40 ± 9.19	0.721
Systolic arterial pressure, mm	110.83 ± 11.14	105.00 ± 9.46	0.241
Mitral inflow E wave, m/s	0.88 ± 0.09	0.83 ± 0.12	0.299
Mitral inflow A wave, m/s	0.60 ± 0.06	0.55 ± 0.08	0.212
Ratio of E to A	1.48 ± 0.24	1.51 ± 0.26	0.775
LVOT velocity, m/s	**1.25 ± 0.05**	1.10 ± 0.31	**0.020**
LVOT VTI, cm	**24.45 ± 1.50**	20.43 ± 3.53	**0.006**
IVCT, ms	65.33 ± 9.44	65.84 ± 13.24	0.841
IVRT, ms	71.17 ± 4.26	62.88 ± 11.14	0.068

Bold text highlights statistically significant values. BD: Behçet’s disease; Ao: aorta, TAPSE: tricuspid annular plane systolic excursion; LVM: left ventricular mass; LVMI: left ventricular mass index; LAtr: left atrium; IVST-D: interventricular septum thickness at end-diastole; IVST-S: interventricular septum thickness at end-systole; LVID-D: left ventricular internal dimension at end-diastole; LVID-S: left ventricular internal dimension at systole; LVPWT-D: left ventricular posterior wall thickness in diastole; LVPWT-S: left ventricular posterior wall thickness in systole; LVOT: left ventricle outflow tract; VTI: velocity time integral; IVCT: isovolumetric contraction time; IVRT: isovolumetric relaxation time.

### Carotid intima-media thickness

**Figure 1.**
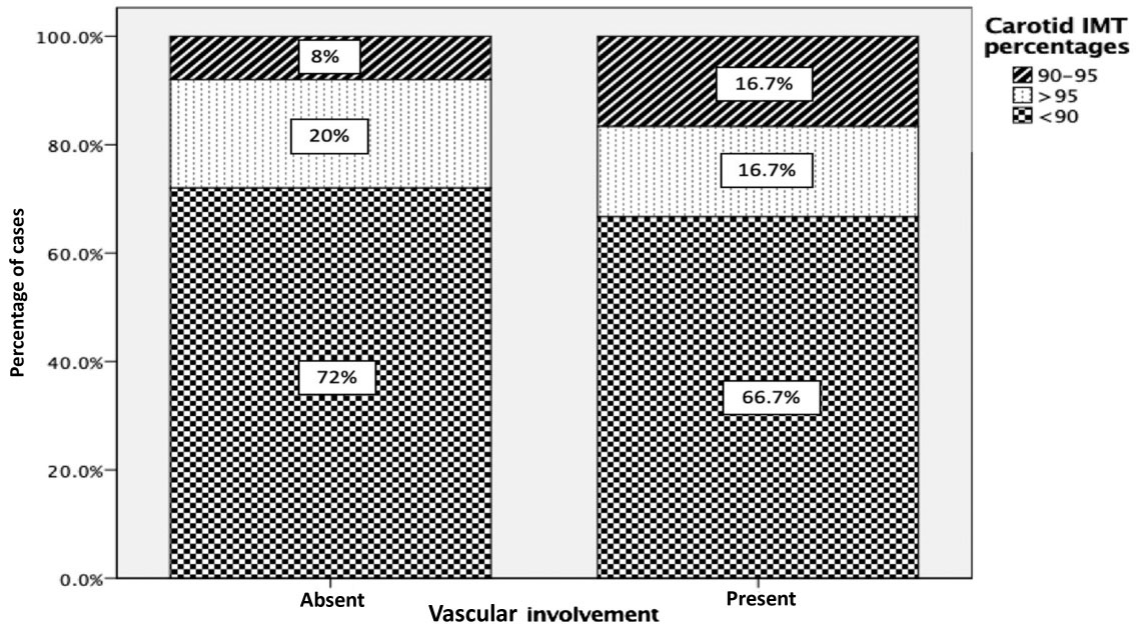
The prevalence of abnormal carotid IMT in patients with vascular involvement compared with patients without vascular involvement (33.3% *vs* 28.0% *P* = 0.798). carotid IMT: carotid intima-media thickness

The cIMT values of the right and left common carotid arteries in BD patients with vascular involvement (0.41 ± 0.5 mm and 0.44 ± 0.7 mm) respectively, and in BD patients without vascular involvement (0.42 ± 0.6 mm and 0.42 ± 0.7 mm) respectively, were not significantly different (Table 4). The prevalence of elevated cIMT (>90th percentile) in patients with and without vascular involvement was statistically similar (33.3% *vs* 28.0% *P* = 0.798) (Fig. 1). cIMT values in BD patients with hypertension (0.50 ± 0.7 mm) were significantly higher than in BD patients without hypertension (0.41 ± 0.5 mm) (*P* = 0.019). Spearman correlation analyses were performed to find the parameters associated with cIMT. cIMT values were positively correlated only with the body mass (*r* = 0.465, *P* = 0.008), height (*r* = 0.364, *P* = 0.044), and left ventricular mass S.D. score (*r* = 0.395; *P* = 0.028). However, they were not correlated with disease duration, age at onset, or age at diagnosis.

**Table 4.** Carotid intima media thickness results of patients with Behçet’s disease according to vascular involvement

Parameters	Vascular involvement		*P*-value
	Yes (*n* = 6)	No (*n* = 25)	
cIMT, mm	0.43 ± 0.5	0.42 ± 0.7	0.841
cIMT S.D. score for age	0.70 ± 1.08	0.68 ± 1.33	0.764
cIMT S.D. score for height	0.78 ± 0.99	0.68 ± 1.28	0.756

cIMT: carotid intima-media thickness.

## Discussion

BD is a very heterogeneous disease in itself and may involve various systems and organs. Patients with BD experience long-term morbidity and even mortality caused by different forms of cardiovascular diseases [23]. Our aim was to find out whether paediatric BD patients with and without vascular involvement had the same risk for cardiovascular comorbidity independent of the contribution of traditional risk factors. In the present study, we showed that 10 patients (34.4%) had abnormal ABPM. However, the carotid IMT and 24-hour ABPM results were not significantly different in paediatric BD patients with and without vascular involvement. In the echocardiographic evaluation, the velocity and velocity time integral of the left ventricle outflow tract differed significantly between the two groups. These findings suggest that paediatric BD patients with vascular involvement may have more increased arterial stiffness compared with BD patients without vascular involvement.

In our study, BD patients with vascular involvement had higher levels of VLDL and triglyceride and lower levels of HDL. Furthermore, although, all patients had inactive disease at the time of study recruiting, patients with vascular involvement had significantly higher levels of CRP. One of the most common causes of mortality in BD is associated with vascular involvement, and it merits more intense CS and immunosuppressive therapy [24]. It is well known that CS therapy may increase total cholesterol and its modifications. Furthermore, previous studies have reported that patients with systemic vasculitis have decreased levels of HDL cholesterol [25]. Increased levels of lipids may have a proinflammatory effect on the vessel wall and contribute to the increased levels of CRP [26]. Therefore, the impaired lipid profile and higher levels of CRP in our patients with vascular involvement may be attributed both to the inflammatory background of BD and possibly to the CS and immunosuppressive therapy.

Atherosclerosis is a chronic inflammatory disease of the arterial intima. Even in children, fatty streaks (which are premature lesions of atherosclerosis) may be present. In the presence of triggering factors, the fatty streak may progress to a complicated atherosclerotic plaque later in life. Systemic inflammatory diseases and increased production of CRP may also accelerate premature atherosclerosis, as has been shown in SLE, RA, granulomatosis with polyangiitis, and Takayasu arteritis [26, 27]. However, there is conflicting evidence regarding the issue of early atherosclerosis in BD [28]. Frequency of coronary atherosclerosis was found to be relatively low even in young male BD patients with major vessel disease [29]. On the other hand, a recent meta-analysis revealed that BD patients had overall thicker carotid intima-media compared with control groups [28].

Considering the inflammatory damage to the vessel wall in patients with BD vasculitis, it is expected that these patients were more at risk of premature atherosclerosis compared with BD patients without vascular involvement. Venous wall thickness (VWT) in lower extremities was shown by Alibaz-Oner *et al.* to be increased in adult BD patients, and these authors suggested that VWT can be used as a diagnostic tool for BD [30]. Recently, Atalay *et al.* evaluated VWT in the lower extremities in paediatric BD and found that increased VWT was present not only in BD patients with vascular involvement but also in those without [31]. Similarly, Caliskan *et al.* showed that IMT results were similar in adult BD patients with and without vascular involvement [32]. In our study, the prevalence of abnormal cIMT in patients with and without vascular involvement was statistically similar, as well. These results may reflect the presence of ongoing subclinical vessel wall inflammation in all BD patients, regardless of vascular involvement.

We also screened all patients with routine echocardiography and aimed to determine whether there was a clinical difference in terms of early cardiac manifestations in BD patients with and without vascular involvement. The velocity and velocity time integral of the left ventricle outflow tract were significantly increased in the patients with vasculitis. Several authors have demonstrated that arterial stiffness is increased in BD. Yılmaz *et al.* reported that patients with active systemic BD involving two or more organs had advanced arterial stiffness [33]. Ikonomidis *et al.* showed that BD patients had decreased aortic elastic properties and left ventricular diastolic function compared with control groups. Furthermore, they showed that BD patients with vascular involvement had higher isovolumetric relaxation time [34]. It would be reasonable to associate vascular BD with the abnormalities in the echocardiography observed in our study. Vasculitis of the vasa vasorum may cause fibrosis, degradation of the elastin within the aortic wall, and lead to the increased arterial stiffness.

A recent meta-analysis demonstrated a significant association between BD and hypertension [35]. In the present study, we also found that 10 patients (34.4%) had abnormal ABPM. Seven patients were non-dippers (24.1%), and hypertension was detected in 5 BD (17.2%, 5/29) patients, despite having no known preexisting hypertension. Moreover, cIMT values in BD patients with hypertension were significantly higher than in BD patients without hypertension. When comparing BD patients with and without vascular involvement the frequencies of abnormal ABPM, non-dipping and hypertension were not significantly different. Our results showed that the use of ABPM in patients with BD allows us to diagnose hypertension earlier and protect them from the increased risk of atherosclerosis.

Our study has several limitations. First, we did not have a healthy control group for comparison. Second, due to the rarity of the disease in childhood, the number of patients with vascular involvement was relatively small. Third, the findings of our study might have been influenced by the different immunosuppressive treatment strategies these patients were receiving.

In conclusion, although paediatric BD patients with vascular involvement may tend to be more predisposed to cardiovascular comorbidity, our findings suggest that there is ongoing subclinical vessel wall inflammation even in patients without vascular involvement. BD has unique features, with significant inflammation in all organ systems, and these patients should be evaluated for cardiovascular risk factors regardless of the involved systems. We suggest that ABPM may accurately identify hypertension and increased cardiovascular risk in BD patients. We need long-term follow-up studies in larger groups to firmly assess the risk of future cardiovascular comorbidities in paediatric-onset BD.

## Data Availability

The data underlying this article will be shared on reasonable request to the corresponding author.

## References

[1] Alpsoy E , Donmez L, Onder M et al Clinical features and natural course of Behcet's disease in 661 cases: a multicentre study. Br J Dermatol 2007;157:901–6. 17711526 10.1111/j.1365-2133.2007.08116.x

[2] Jennette JC , Falk RJ, Bacon PA et al 2012 revised International Chapel Hill Consensus conference nomenclature of vasculitides. Arthritis Rheum 2013;65:1–11. 23045170 10.1002/art.37715

[3] Kone-Paut I. Behcet's disease in children, an overview. Pediatr Rheumatol Online J 2016;14:10. 26887984 10.1186/s12969-016-0070-z PMC4758175

[4] Desbois A-C , Wechsler B, Cluzel P et al Cardiovascular involvement in Behcet's disease]. Rev Med Interne 2014;35:103–11. 24434015 10.1016/j.revmed.2013.12.002

[5] Juonala M , Viikari JSA, Laitinen T et al Interrelations between brachial endothelial function and carotid intima-media thickness in young adults: the cardiovascular risk in young Finns study. Circulation 2004;110:2918–23. 15505080 10.1161/01.CIR.0000147540.88559.00

[6] Corrado E et al Relationship between endothelial dysfunction, intima media thickness and cardiovascular risk factors in asymptomatic subjects. Int Angiol 2005;24:52–8. 15876999

[7] Chambers JC , Haskard DO, Kooner JS. Vascular endothelial function and oxidative stress mechanisms in patients with Behcet's syndrome. J Am Coll Cardiol 2001;37:517–20. 11216972 10.1016/s0735-1097(00)01137-2

[8] Protogerou A , Lekakis J, Stamatelopoulos K et al Arterial wall characteristics in patients with Adamantiades-Behcet's disease. Adv Exp Med Biol 2003;528:399–404. 12918733 10.1007/0-306-48382-3_82

[9] Kayikcioglu M et al Endothelial functions in Behcet's disease. Rheumatol Int 2006;26:304–8. 15739096 10.1007/s00296-005-0590-1

[10] Keser G et al Increased thickness of the carotid artery intima-media assessed by ultrasonography in Behcet's disease. Clin Exp Rheumatol 2005;23(4 Suppl 38):S71–6. 16273769

[11] Geri G , Wechsler B, Thi Huong DL et al Spectrum of cardiac lesions in Behcet disease: a series of 52 patients and review of the literature. Medicine (Baltimore) 2012;91:25–34. 22198500 10.1097/MD.0b013e3182428f49

[12] Kechida M , Salah S, Kahloun R et al Cardiac and vascular complications of Behcet disease in the Tunisian context: clinical characteristics and predictive factors. Adv Rheumatol 2018;58:32. 30657088 10.1186/s42358-018-0032-x

[13] Yavne Y , Tiosano S, Watad A et al Investigating the link between ischemic heart disease and Behcet's disease: a cross-sectional analysis. Int J Cardiol 2017;241:41–5. 28285799 10.1016/j.ijcard.2017.02.135

[14] Sun BJ , Park J-H, Yoo S-J et al Intrinsic changes of left ventricular function in patients with Behcet disease and comparison according to systemic disease activity. Echocardiography 2018;35:809–16. 29451950 10.1111/echo.13844

[15] Barnado A , Carroll RJ, Casey C et al Phenome-wide association study identifies marked increased in burden of comorbidities in African Americans with systemic lupus erythematosus. Arthritis Res Ther 2018;20:69. 29636090 10.1186/s13075-018-1561-8 PMC5894248

[16] Magder LS , Petri M. Incidence of and risk factors for adverse cardiovascular events among patients with systemic lupus erythematosus. Am J Epidemiol 2012;176:708–19. 23024137 10.1093/aje/kws130 PMC3571250

[17] Manzi S , Meilahn EN, Rairie JE et al Age-specific incidence rates of myocardial infarction and angina in women with systemic lupus erythematosus: comparison with the Framingham Study. Am J Epidemiol 1997;145:408–15. 9048514 10.1093/oxfordjournals.aje.a009122

[18] Koné-Paut I , Shahram F, Darce-Bello M et al Consensus classification criteria for paediatric Behcet's disease from a prospective observational cohort: PEDBD. Ann Rheum Dis 2016;75:958–64. 26698843 10.1136/annrheumdis-2015-208491

[19] Lawton G , Bhakta BB, Chamberlain MA et al The Behcet's disease activity index. Rheumatology (Oxford) 2004;43:73–8. 12890862 10.1093/rheumatology/keg453

[20] Wühl E , Witte K, Soergel M et al Distribution of 24-h ambulatory blood pressure in children: normalized reference values and role of body dimensions. J Hypertens 2002;20:1995–2007. 12359978 10.1097/00004872-200210000-00019

[21] Doyon A , Kracht D, Bayazit AK et al; 4C Study Consortium. Carotid artery intima-media thickness and distensibility in children and adolescents: reference values and role of body dimensions. Hypertension 2013;62:550–6. 23817494 10.1161/HYPERTENSIONAHA.113.01297

[22] Lang RM , Bierig M, Devereux RB et al; European Association of Echocardiography. Recommendations for chamber quantification: a report from the American Society of Echocardiography's Guidelines and Standards Committee and the Chamber Quantification Writing Group, developed in conjunction with the European Association of Echocardiography, a branch of the European Society of Cardiology. J Am Soc Echocardiogr 2005;18:1440–63. 16376782 10.1016/j.echo.2005.10.005

[23] Silveira LH. Cardiovascular manifestations of systemic vasculitides. Curr Rheumatol Rep 2020;22:72. 32856161 10.1007/s11926-020-00952-1

[24] Tascilar K , Melikoglu M, Ugurlu S et al Vascular involvement in Behcet's syndrome: a retrospective analysis of associations and the time course. Rheumatology (Oxford) 2014;53:2018–22. 24907156 10.1093/rheumatology/keu233

[25] Cohen Tervaert JW. Cholesterol and modifications of cholesterol in rheumatic disorders. In: Hoffman GS, Weyand CM. Goronzy J.J., Langford CA, editors. Inflammatory diseases of blood vessels. 2nd edn. Wiley-Blackwell, 2012: 475–83.

[26] Cohen Tervaert JW. Cardiovascular disease due to accelerated atherosclerosis in systemic vasculitides. Best Pract Res Clin Rheumatol 2013;27:33–44. 23507055 10.1016/j.berh.2012.12.004

[27] Tervaert JW. Translational mini-review series on immunology of vascular disease: accelerated atherosclerosis in vasculitis. Clin Exp Immunol 2009;156:377–85. 19309350 10.1111/j.1365-2249.2009.03885.x PMC2691964

[28] Merashli M , Ster IC, Ames PR. Subclinical atherosclerosis in Behcet's disease: a systematic review and meta-analysis. Semin Arthritis Rheum 2016;45:502–10. 26239908 10.1016/j.semarthrit.2015.06.018

[29] Seyahi E , Memisoglu E, Hamuryudan V et al Coronary atherosclerosis in Behcet's syndrome: a pilot study using electron-beam computed tomography. Rheumatology (Oxford) 2004;43:1448–50. 15501997 10.1093/rheumatology/keh359

[30] Alibaz-Oner F , Ergelen R, Mutis A et al Venous vessel wall thickness in lower extremity is increased in male patients with Behcet's disease. Clin Rheumatol 2019;38:1447–51. 30758790 10.1007/s10067-019-04470-z

[31] Atalay E , Oguz B, Sener S et al A new tool supporting the diagnosis of childhood-onset Behcet's disease: venous wall thickness. Rheumatology (Oxford) 2023;62:SI181–SI188. 35640152 10.1093/rheumatology/keac314

[32] Caliskan M , Gullu H, Yilmaz S et al Cardiovascular prognostic value of vascular involvement in Behcet's disease. Int J Cardiol 2008;125:428–30. 17408778 10.1016/j.ijcard.2007.01.057

[33] Yilmaz S , Celik G, Esmen SE. Assessment of arterial stiffness in patients with inactive and active Behcet's disease. Scand J Rheumatol 2014;43:63–9. 24015673 10.3109/03009742.2013.809787

[34] Ikonomidis I , Lekakis J, Stamatelopoulos K et al Aortic elastic properties and left ventricular diastolic function in patients with Adamantiades-Behcet's disease. J Am Coll Cardiol 2004;43:1075–81. 15028369 10.1016/j.jacc.2003.10.042

[35] Chen T , Shao X, Li H et al Association of Behcet's disease with the risk of metabolic syndrome and its components: a systematic review and meta-analysis. Clin Exp Med 2023;23:2855–66. 36939969 10.1007/s10238-023-01044-x PMC10543763

